# Mobile microscopy and telemedicine platform assisted by deep learning for the quantification of *Trichuris trichiura* infection

**DOI:** 10.1371/journal.pntd.0009677

**Published:** 2021-09-07

**Authors:** Elena Dacal, David Bermejo-Peláez, Lin Lin, Elisa Álamo, Daniel Cuadrado, Álvaro Martínez, Adriana Mousa, María Postigo, Alicia Soto, Endre Sukosd, Alexander Vladimirov, Charles Mwandawiro, Paul Gichuki, Nana Aba Williams, José Muñoz, Stella Kepha, Miguel Luengo-Oroz

**Affiliations:** 1 Spotlab, Madrid, Spain; 2 Biomedical Image Technologies, ETSI Telecomunicación, Universidad Politécnica de Madrid, Madrid, Spain; 3 Eastern and Southern Africa Center for International Parasite Control (ESACIPAC), Kenya Medical Research Institute (KEMRI), Nairobi, Kenya; 4 Barcelona Institute for Global Health (ISGlobal), Hospital Clínic-Universitat de Barcelona, Barcelona, Spain; Swiss Tropical and Public Health Institute, SWITZERLAND

## Abstract

Soil-transmitted helminths (STH) are the most prevalent pathogens among the group of neglected tropical diseases (NTDs). The Kato-Katz technique is the diagnosis method recommended by the World Health Organization (WHO) although it often presents a decreased sensitivity in low transmission settings and it is labour intensive. Visual reading of Kato-Katz preparations requires the samples to be analyzed in a short period of time since its preparation. Digitizing the samples could provide a solution which allows to store the samples in a digital database and perform remote analysis. Artificial intelligence (AI) methods based on digitized samples can support diagnosis by performing an objective and automatic quantification of disease infection. In this work, we propose an end-to-end pipeline for microscopy image digitization and automatic analysis of digitized images of STH. Our solution includes (a) a digitization system based on a mobile app that digitizes microscope samples using a 3D printed microscope adapter, (b) a telemedicine platform for remote analysis and labelling, and (c) novel deep learning algorithms for automatic assessment and quantification of parasitological infections by STH. The deep learning algorithm has been trained and tested on 51 slides of stool samples containing 949 *Trichuris* spp. eggs from 6 different subjects. The algorithm evaluation was performed using a cross-validation strategy, obtaining a mean precision of 98.44% and a mean recall of 80.94%. The results also proved the potential of generalization capability of the method at identifying different types of helminth eggs. Additionally, the AI-assisted quantification of STH based on digitized samples has been compared to the one performed using conventional microscopy, showing a good agreement between measurements. In conclusion, this work has presented a comprehensive pipeline using smartphone-assisted microscopy. It is integrated with a telemedicine platform for automatic image analysis and quantification of STH infection using AI models.

## Author summary

Soil-transmitted helminths (STH), including hookworm, *Ascaris lumbricoides* and *Trichuris trichiura*, are common intestinal infections in low-income countries. Global estimates indicate that more than 1.5 billion people are infected with at least one STH species. They cause anaemia, gastro-intestinal problems, tiredness amongst other symptoms. Diagnosis of STH infection is mainly performed by analyzing stool samples under the microscope using the so-called Kato-Katz technique. However, the analysis of Kato-Katz samples, which is usually performed by microscopy experts, is a subjective procedure based on visual inspection of the samples and requires to be done in a short period of time since the sample preparation. In this work we proposed a novel system to digitize the microscopy samples using an affordable 3D-printed adapter and smartphones. Digitized images were uploaded to a telemedicine platform enabling remote diagnosis. Additionally, the digitized images were automatically analyzed by an Artificial Intelligence (AI) algorithm which was fully-integrated in the telemedicine platform, performing an automatic and objective count of different types of STH parasites (*A*. *lumbricoides* and *T*. *trichiura)*.

## 1. Introduction

Soil-transmitted helminths (STH), which include hookworms (*Ancylostoma duodenale* and *Necator americanus*), roundworm (*Ascaris lumbricoides*), and whipworm (*Trichuris trichiura*) are the most prevalent pathogens among the group of neglected tropical diseases (NTDs) and occur predominantly in low- and middle-income tropical and subtropical countries [1]. Globally STH affects more than 1.5 billion people, causing a loss of more than 3 million disability adjusted life years (DALYs) [2]. The World Health Organization (WHO) 2030 Roadmap for NTDs set out strategies for STH control that focused on the elimination of the disease caused by these parasites as a public health problem [3].

In many endemic countries, the STH control strategy is implemented through targeted mass drug administration (MDA), using the anthelmintic drugs benzimidazole (BZ), albendazole, or mebendazole [4]. The diagnostic method recommended by WHO is Kato-Katz, a laboratory method for preparing human stool samples in a microscope smear using a small spatula and slide template. It allows a standardized amount of faeces to be examined under a microscope and quantify STH infection [5,6]. Kato-Katz is generally more sensitive than other microscopic methods such as McMaster, formol-ether concentration, and direct microscopy in high transmission settings, and it requires limited equipment and is easy to perform in low resource settings [7]. However, it often presents a decreased sensitivity in low-transmission settings.

One of the main disadvantages of the Kato-Katz technique is the necessity to read samples within 30 minutes from preparation as eggs tend to disappear or hatch, specially those of hookworms, and thus considerably reducing the sensitivity of this technique and even highly trained microscopists can misidentify species or give inconsistent results [8,9]. Digitizing the samples at the right time (within 30 to 60 minutes after slide preparation) could provide a solution that allows digital storage of the sample images and further analysis at any moment in time. Different digitization devices have been tested allowing remote diagnosis and second opinion [10]. Additionally, during the last years, the potential of the use of smartphones in the diagnosis of parasitic diseases has been highlighted, and there have been proposed smartphone-assisted systems for microscopy [11]. A further step that could increase performance and remove subjectivity of microscopic techniques is the possibility of implementing artificial intelligence (AI) algorithms for the automatic detection and quantification of these parasites on digitized image samples. This would be a major advance in the diagnosis and control of these diseases and its implementation would not disrupt the laboratories normal workflow since the basis of diagnosis is still microscopy and the technique used is the Kato-Katz.

AI-based technologies are rapidly evolving into applicable medical solutions and are actually revolutionizing the field [12]. However, only a few studies have made effort to meet rigorous standards to be approved by regulatory institutions such as the Food and Drug Administration (FDA) [13]. Most of these approved technologies were developed for the fields of radiology, cardiology, and internal medicine. However, AI systems also have the potential to be applied to enable a rapid and objective diagnosis of NTDs and to enable the delivery of public health in low- and middle-income countries. In this context, a special effort has to be made for the application of AI methods in such diseases as has been recommended by the WHO [14].

Several approaches for computer-aided analysis of helminth eggs detection and classification using AI have been investigated in the last few years. Alva *et al*. proposed the use of hand-crafted features along with a multivariate logistic regression for intestinal parasites classification [15]. Image analysis techniques based on morphological operations have also been proposed to identify pathogenic helminth eggs, although their authors found limitations in their use in samples with very high solids content due to the large amount of debris and objects that interfere with the precise identification of eggs [16]. Another notable recent deep learning-based approach used a large fecal database with over 1122 patients including 22440 images for the identification of visible components in faeces, including blood and epithelial cells, as well as STH eggs, proposing the so-called FecalNet [17]. This work proved the potential of using these methods for the automatic analysis of stool samples using conventional microscopy images. Holmstrom et al. proposed to acquire microscopy images with a portable scan connected to a laptop and used a two-stage sequential algorithm where candidates previously proposed by the first algorithm are classified as any type of helminth egg, and obtained promising results despite their limited number of training samples [18]. The use of deep learning-based object detection methods for automatic analysis and detection of helminth eggs in images acquired with smartphone-compatible microscopy attachments has already been tested [19]. This work achieved sensitivity comparable to standard microscopy when detecting *Ascaris* spp. but showed low performance in the identification of *Trichuris* spp. This is probably caused by the use of a cheap smartphone-compatible microscopy attachment (a magnification endoscope; USB Video Class, UVC) where the light source comes from the same direction as the camera. This produces images with insufficient quality specially for *Trichuris* spp. which have thinner and more translucent membranes. Previous approaches might disrupt the usual laboratory workflow as they do not use conventional microscopes.

The objective of this study is to propose and develop an end-to-end system for remote and automatic detection and quantification of STH primarily for the detection of *T*. *trichiura*, based on digitized microscopy images acquired with a 3D printed adapter together with a smartphone and AI methods.

## 2. Materials and methods

### 2.1. Ethics statement

Ethical approval was obtained from the Kenya Medical Research Institute (KEMRI) Ethics Review Committee (SERU 3873). Prior to enrolment, written informed consent was obtained from all participating individuals and parents/guardians of children, all individual adults in the households and assent from children aged 13 years and above. All the individuals found positive for STH were treated with albendazole by community health workers.

### 2.2. Data and samples

**Fig 1:**
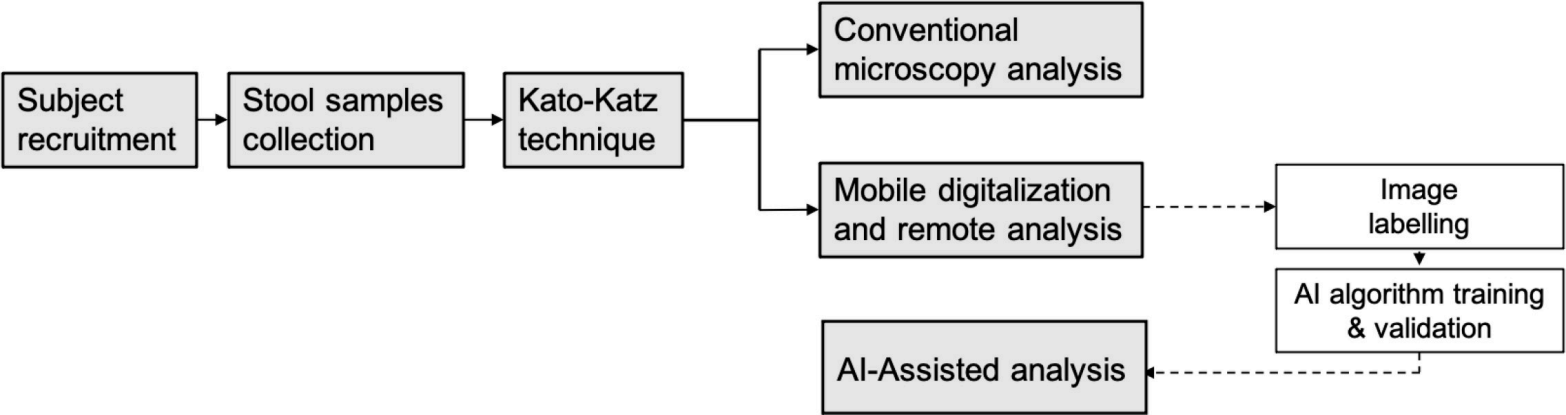
Schematic representation of study design and experimental workflow.

In this work, we present a complete end-to-end pipeline from stool sample collection to automatic analysis of the samples for the identification of STH parasites. **Fig 1** schematizes the proposed end-to-end workflow, where samples collected and prepared from different subjects were digitized and uploaded to a telemedicine platform for remote analysis. Additionally, the digitized samples were labelled using the telemedicine platform and used for training AI algorithms in order to perform an automatic analysis for future incoming digitized samples. All the prepared samples were also analyzed during the process using conventional microscopy methods for comparison purposes.

Stool was collected from study participants from Kwale county (south coast of Kenya) who were part of a follow-up study related to exploring the presence of *T*. *trichiura* infection. All stool samples were transferred to the laboratory within four hours after collection and processed using the Kato-Katz thick smear method (using a template that allows one to put 41.7 mg of stool sample on the slide) and analyzed by conventional microscopy on the same day to identify and quantify the presence of STH eggs. This analysis was carried out by a total of 3 independent technicians and all samples were analyzed by at least two of them. We digitized using the proposed digitization pipeline samples from 12 subjects (6 positive and 6 negatives). These digitized samples were used for the evaluation of the remote analysis system, as well as to train and evaluate the AI algorithm. From the 6 positive subjects, 5 were only positive for *Trichuris* spp. and one was co-infected by *Trichuris* spp. and *Ascaris* spp. For each positive stool sample, 7 slides were prepared, while for negative stool samples 1 or 2 slides were prepared.

### 2.3 Digitization pipeline of clinical samples

**Fig 2:**
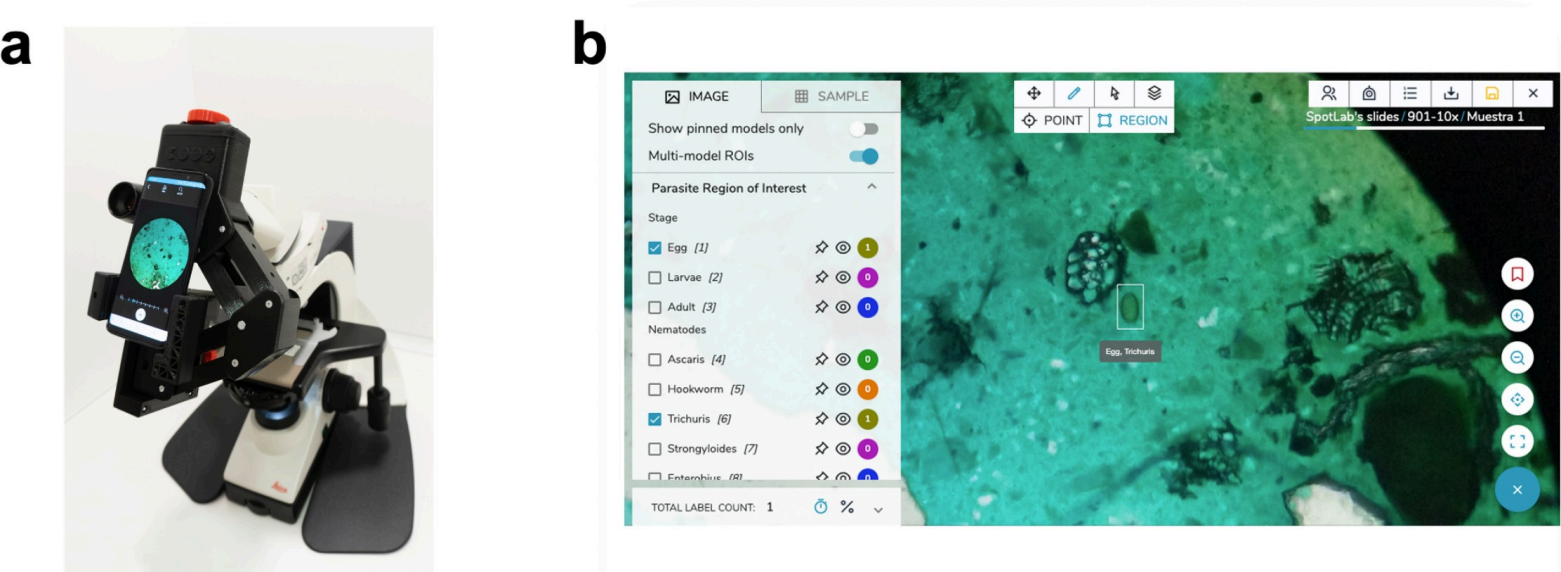
(A) 3D-printed adapter used for attaching a smartphone to a conventional microscope and digitizing samples through a mobile app. (B) Telemedicine web platform for viewing and remote analysis of the digitized samples performing a manual annotation. Note: The web platform in Fig 2B has been developed by the authors.

The proposed digitization system uses a 3D printed device that allows coupling a mobile phone with a conventional optical microscope by aligning the smartphone camera with the objective of the microscope to acquire images. This adapter (approximately 0.5 kg, with a compact design measuring 31x16x12 cm) has the potential to convert any conventional microscope into a digital microscope. It should be noted that the working method when using the adapter remains the same as in the conventional way, and the user still needs to move the microscope eyepiece navigating through the sample. The smartphone, which is placed in the adapter, uses an Android mobile app specifically developed and customized for fast and easy digitization and sharing of digitized microscopy images (**Fig 2A**). 51 Kato-Katz slides were digitized with a 10x objective (100x total magnification) using two different smartphone models (Xiaomi Pocophone F1 and Bq Aquaris X2) by attaching the 3D printed device to the ocular of a conventional light microscope (Leica DM-2000). It should be noted that we used two different smartphone models during the digitization process in order to introduce potential variability in our image database due to the use of different devices. All microscopy fields in which helminth eggs were present or suspected were digitized. In addition, visually confirmed negative images were also acquired for both positive and negative subjects. Images were acquired in the JPG format with a resolution of 12 Mpx through the mobile application.

### 2.4. Telemedicine platform for remote analysis

All acquired images were transferred from the smartphone to a telemedicine platform via the mobile network, where the images are stored and presented in an easy-to-use dashboard that allows their visualization, management, and labelling (see **Fig 2B**). In this web platform, standard clinical and analysis protocols were translated into digital tasks that were adapted to the clinical case and disease under study (see the analysis panel shown in the left part of the screenshot presented in **Fig 2B**).

All acquired images were labelled through the telemedicine platform by one expert using a customized procedure for analysing stool samples which allows to tag parasites that can be seen in the images and thus quantify parasitic infection. The annotation protocol was based on the placement of bounding boxes around the identified parasites. The image database, together with the label data, can be accessed by other professionals and coupled with other support platforms for diagnostic assistance.

### 2.5. Artificial intelligence algorithm

#### 2.5.1. Network architecture

The proposed approach for helminth eggs identification relies on a detection algorithm based on Convolutional Neural Networks (CNN). Most CNN-based detection algorithms are designed to perform at the same time both the object localization task, which determines where objects are located in a given image, and the object classification task, which determines which particular category each previously located object belongs to.

In this paper, Single-Shot Detection (SSD) architecture [20] together with the backbone MobileNet network [21] for feature extraction were used. Our choice to use a SSD-MobileNet architecture is mainly due to the relative simplicity of the architectures which are specially designed to be easily integrated into embedded vision applications in an efficient way. The model was initialized with pretrained weights on the COCO Image database [22]. We used the RMSprop optimizer with an exponential decay learning rate to minimize the total loss function, which was calculated as the sum of a sigmoid cross entropy loss for object classification and smooth L1 loss for object localization. We also employed early stopping technique, where the training finishes before overfitting begins by stopping the training process when the error on the validation set does not decrease for a predefined number of steps.

#### 2.5.2 Training dataset generation

The training dataset was generated by extracting 512x512 pixels image patches around the location of labels which were placed manually by the experts on helminths. The size of the image patches was selected based on a balance between the relative size of the objects in relation to the size of the image patches and the computational cost needed at inference time, as a small size of image patches increases the number of patches to be processed at test time using the sliding window procedure (see Section 3.3.4).

Additionally, and for the purpose of augmenting the size of the training dataset, we randomly selected 512x512 pixels image patches around the manually placed bounding boxes multiple times, always ensuring that all labeled objects were fully covered. This was done so that each object could appear in different locations within the image patch, and different context environments were captured. Moreover, additional augmentation was conducted by applying on-the-fly random flip and rotation transformations during training.

The framework used for training the proposed method was based on Tensorflow Object Detection API using a cloud computing environment with a GPU Nvidia Tesla K80 12GB. The time required to train the algorithm using the described hardware was approximately 3 hours.

## 3. Experiments and results

To evaluate and validate the entire proposed pipeline for digitizing and automatically assessing microscopy images, we first evaluate the quality of annotations on digitized images and analyze whether they can serve as ground truth for training the AI algorithm. This evaluation was performed using the analysis based on a conventional procedure as reference. Subsequently, we evaluated the performance of the AI model for the detection of helminth eggs.

### 3.1. Generation and evaluation of ground-truth annotations

Bounding boxes around the identified parasites annotated through the telemedicine platform were used as ground truth for training the AI algorithm. From the 51 Kato-Katz slides, a total of 1508 image fields were digitized and uploaded to the telemedicine platform, analyzed and labeled by a microscopist expert. Manual revision of these images resulted in a total of 797 positive images for at least one STH and 711 negative images.

**Table 1** summarizes the digitized samples from all positive patients where a total of 949 *Trichuris* spp. egg labels were identified. It should be noted that patient number 6 had a biparasite infection for *Trichuris* spp. and *Ascaris* spp., obtaining additional 4296 labels for *Ascaris* spp. eggs. Additionally, as expected, all 10 digitized slides from the 6 negative subjects obtained a negative result where no eggs were found in the images.

**Table 1:** Database of digitized Kato-Katz slide samples from the 6 positive patients. *Patient number 5 had 6 slides instead of 7 because one of them broke during its handling.

	Patient	N° of slides	N° of (+) images	N° of (-) images	Total number of images	N° *Trichuris* spp. Eggs
**Positive subjects**	**1**	7	94	39	133	148
	**2**	7	39	15	54	47
	**3**	7	50	21	71	74
	**4**	7	28	203	231	30
	**5**	6*	44	10	54	47
	**6**	7	542	13	555	603
**Total**		**41**	**797**	**301**	**1098**	**949**

Once we obtained the ground truth for AI models by manually annotating acquired images, we evaluated its quality to study all possible biases introduced during the digitization step. In this way, the manual egg counting using digitized images was compared to the analysis performed by a conventional microscopy procedure on the same samples.

**Fig 3:**
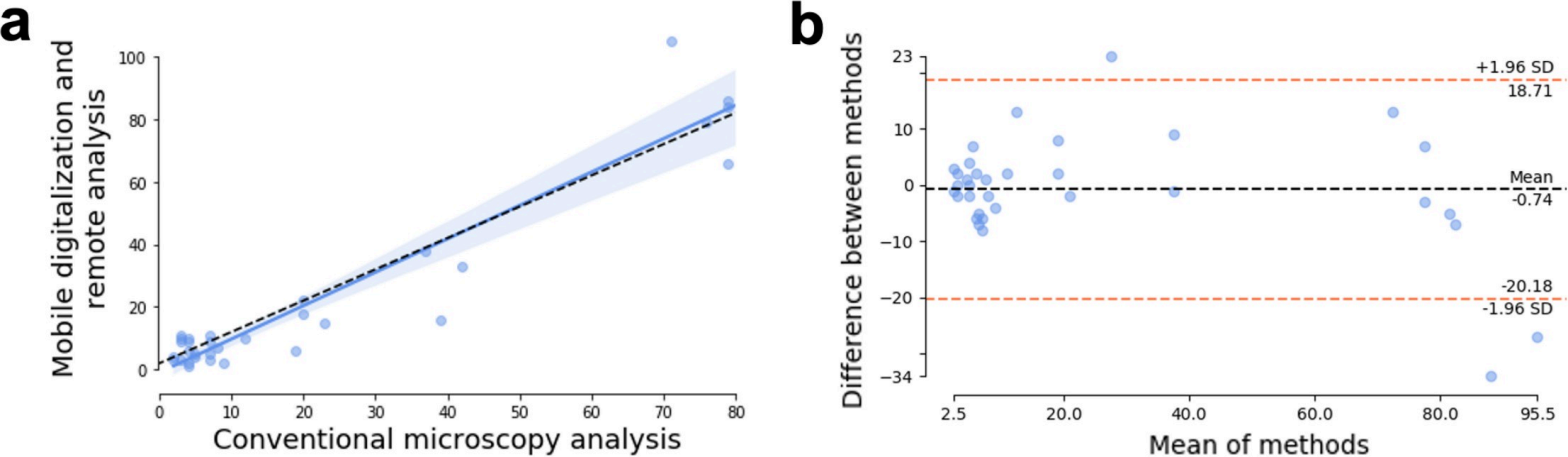
Assessment of the quality of the generated ground truth. (A) Scatterplot of the manual counting using conventional microscopy and using digitized samples. (B) Bland-Altman plot showing the difference between the conventional and digital analyses.

For the comparison of the two measurement methodologies (conventional microscopy and remote analysis of digitized samples which serve as ground truth for training the AI algorithm) we excluded patient number 3 because its slides were not properly preserved and their reading could not be done correctly. To evaluate the quality of the ground truth generated for training AI models, we study the correlation between both egg measurements made by the conventional procedure and based on digitized images. We found a strong correlation between both metrics, with a Pearson correlation coefficient of 0.95 (95% CI:[0.91–0.98], p-value <0.001). (see **Fig 3A**). Bland-Altman analysis [23] was also performed to analyze the goodness of the generated ground truth, and the results showed good agreement between both metrics (mean bias of 0.74 units, see **Fig 3B**). This slight overestimation of egg count observed in the manual labelling of digitized samples compared to the conventional methodology may indicate that when the count and analysis were performed remotely on the telemedicine platform, the expert can perform a more exhaustive job, detecting more eggs than when it is done in the field, and decreasing the number of false negatives.

The results ensure the quality of the generated ground truth based on digitized images of Kato-Katz samples and indicate that they can be used for training AI models.

### 3.2. Artificial intelligence-assisted analysis

#### 3.2.1 Analysis of the model performance

To evaluate the proposed method for *Trichuris* spp. detection, we constructed a leave-one-out cross-validation scheme at the patient level. Thus, the algorithm is applied and tested only on samples belonging to a single subject, using all other subject samples as a training set. This process ensures the test set is completely independent from the training set. It should be noted that as the proposed algorithm was focused on the detection of *Trichuris* spp., the leave-one-out scheme was constructed based only on subjects who were positive for *Trichuris* spp.

To assess the model performance, we considered the precision (P), recall (R), and F-score (F) metrics defined as:

$$P=\frac{TP}{TP+FP},\;R=\frac{TP}{TP+FN},\;F=2\times \frac{P\times R}{P+R}$$

where TP, FP and FN denote the number of true positives, false positives, and false negatives, respectively. TP were defined as both correctly boxed and classified object, false detection was considered as FP and FN was defined as all ground truth objects misdetected by the algorithm or proposed for a wrong label. All the boxes proposed by the algorithm had to have a certainty greater than or equal to 30% to be considered as proposed. The certainty of an object was defined based on the probability given by the algorithm and associated with the predicted label of this object. Correctly proposed and classified boxes were considered as true positive detections when they had an intersection over union (IOU) with the ground truth greater than 30%. Note that the performance metrics were only computed on positive image patches i.e., known to contain at least one helminth egg.

An overview of the results is shown in **Table 2**. The proposed approach showed a mean precision (P) of 98.4%, a mean recall (R) of 80.9% and a mean F-score (F) of 88.5% along all folders within the leave-one-out cross validation scheme.

**Table 2:** Detailed performance of the proposed methodology for the detection of *Trichuris* spp. using a leave-one-one cross-validation scheme at the patient level.

	Training	Testing			
	#T. eggs	#T. eggs	Precision	Recall	F-Score
**Folder 1**	801	148	99.16	79.73	88.39
**Folder 2**	902	47	97.78	93.62	95.65
**Folder 3**	875	74	100.00	66.22	79.68
**Folder 4**	919	30	100.00	80.00	88.89
**Folder 5**	902	47	95.24	85.11	89.89
***Mean*** ***STD***			**98.44** **2.00**	**80.94** **9.96**	**88.50** **5.73**

Note: #T. eggs represent the number of *Trichuris* spp.

Additionally, we wanted to compare the results obtained with the proposed method, which is based on SSD and MobileNet networks, with a model based on FasterRCNN together with a ResNet50 backbone, a more complex and deeper network, which also introduces the concept of residual connections. Our hypothesis is that, although this deeper network may have a higher discriminative power, it needs to be trained with a large amount of training data. The network was trained with the same leave-one-out cross-validation scheme as that used for training the proposed algorithm and obtained a mean precision of 88.5% and a mean recall of 75.1%. It should also be noted that the FasterRCNN-ResNet50 algorithm took longer to train and test compared to the proposed SSD-MobileNet architecture, imposing more computational cost. These results prove that the proposed method outperforms more complex networks, being an optimally cost-effective architecture.

For further validation, we wanted to extend the analysis of the AI algorithm developed by comparing its results at slide level to those obtained by all reference methods, including both conventional microscopy-based analysis and manual remote analysis of digitized samples.

Digitized images from whole slides were divided into 512x512 pixels image patches using a sliding window procedure with an overlap of 64 pixels to ensure all helminth eggs are not cropped within images. All 27 slides coming from all subjects only positive for *Trichuris* spp. that were included in the analysis of Section 3.1 were analyzed by the AI algorithm, and Bland-Altman analysis between automated AI-assisted STH quantification and both manual remote analysis and the one performed by conventional microscopy were assessed.

Bland-Altman analysis showed a good agreement between AI-assisted results and both the conventional microscopy procedure and the manual remote analysis, having a bias of -0.26, 95% confidence interval [-16.78,16.26] when compared to the results obtained with the conventional microscopy method, and a bias of -1.4 units, 95% confidence interval [-9.01,6.20] when compared to the remote analysis of digitized samples.

Additionally, we also executed the AI algorithm in all images extracted from all negative samples. The results showed that only 99 STH eggs were incorrectly detected (false positives) along the 20090 negative images, resulting in a specificity of greater than 99% at the image patch level.

#### 3.2.2 Evaluation of the generalization capability

For further assessment of the proposed approach, we also wanted to test the generalization capability of the proposed methodology at detecting other helminths eggs than *Trichuris* spp. We extended our analysis by training the proposed method with all available subject samples, i.e. including those belonging to the one which were positive for a coinfection of *Trichuris* spp. and *Ascaris* spp. The training and evaluation of the extended version of the proposed method were based on a train-validation-test scheme. Of all available digitized slides from all subjects, 32 were randomly selected for training and validation, while the remaining 9 were used for testing. Both the training sets (70%) and the validation sets (15%) comprised 808 *Trichuris* spp. and 3649 *Ascaris* spp. eggs, while the test set (15%) consisted of 136 *Trichuris* spp. and 647 *Ascaris* spp. eggs. Our interpretation is that the appearance of helminth eggs is independent across slides and therefore the training and testing may be done using images from the same subject as long as they belong to different slides.

Additionally, and in order to increase the discriminating power of the proposed approach and to better distinguish between both helminth eggs and all those structures that may be present on the images which are similar to objects under study, we deployed our trained model on all training slides, identified all false positives objects, labeled them as a separated class (namely artifact class) and constructed a multiclass model including three different classes, namely *Trichuris* spp., *Ascaris* spp. and artifacts. Thus, background objects which are difficult to discern and are confused with real helminth eggs were used as negative examples.

**Table 3** summarizes the results and proves that the proposed model can be extended for the detection of different helminth eggs, obtaining promising results, including a mean precision of 94.36% and a mean recall of 93.08%.

**Table 3:** Overview of the results obtained for the detection of helminth eggs from *Trichuris* spp. and *Ascaris* spp.

	Precision	Recall	F-Score
***Trichuris* spp.**	95.31	89.71	92.43
***Ascaris* spp.**	93.41	96.45	94.91
**Mean**	94.36	93.08	93.97

### 3.3. Built-in AI algorithms: operational deployment on technological platforms

**Fig 4:**
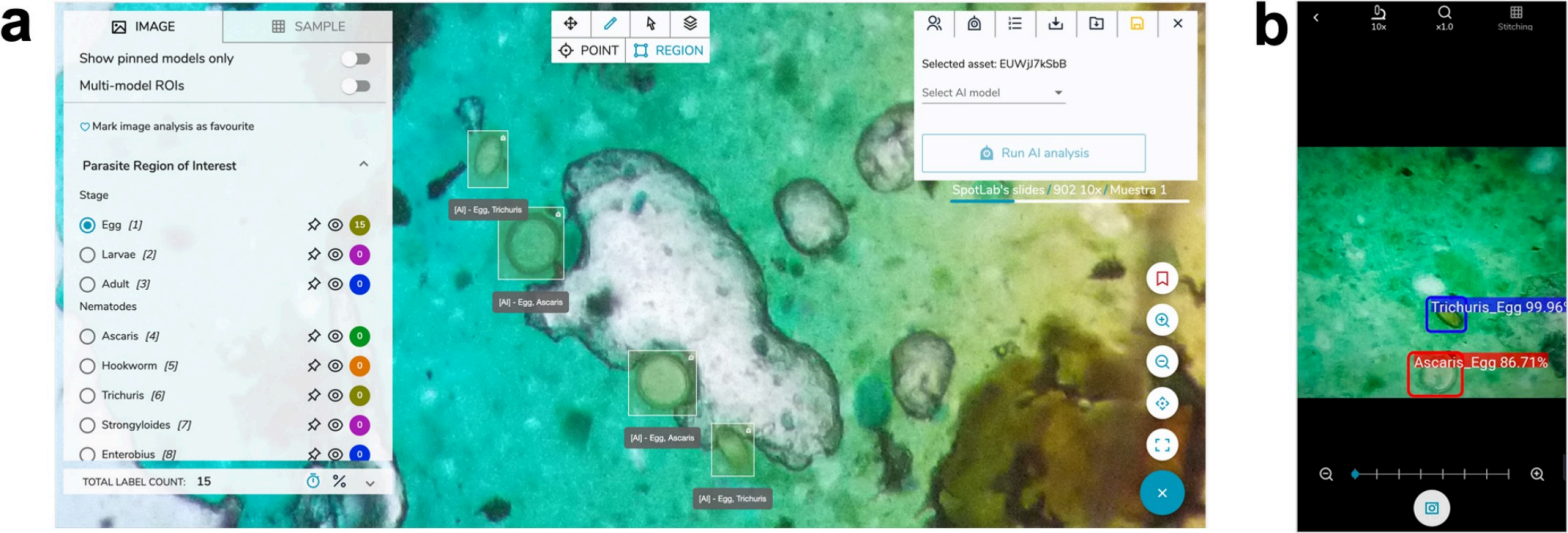
Operational deployment of the AI algorithm on technological platforms. (A) Deployment on the telemedicine platform. (B) Prototype of the deployment on the mobile acquisition app. Note: Both the web platform and the mobile application in Fig 4A and 4B have been developed by the authors.

The AI algorithm developed was integrated into both the telemedicine platform and the acquisition mobile app, allowing the operational deployment of the AI model so that it can be remotely used on demand by the telemedicine platform or even could be executed during acquisition time through the mobile app. **Fig 4** shows the implementation of the AI algorithm on both technological platforms.

The execution of the AI algorithm on the telemedicine platform is done on a cloud computing platform. Each time the images are uploaded, the system recognizes the type of sample based on the image metadata and executes the AI model which will detect the eggs and show them in a visualization environment which is accessed via the web. As an additional possibility of integration of AI models into the clinical workflow, we have created a prototype that embeds the AI model in the acquisition app. While the user examines the sample with the smartphone attached to the microscope, the images captured by the camera are processed in real time, and all STH eggs detected by the AI algorithm locally executed on the smartphone’s hardware are shown in an augmented reality environment (see **Fig 4B**). This could facilitate the identification and counting of STH eggs by the user.

**Table 4** provides a report of the calculated time needed to perform the prediction for a single image patch of 512x512 pixels using different hardware configurations and technological platforms, as well as the average time needed to get a final prediction for a whole digitized image considering that each image is composed of 48 image patches.

**Table 4:** Comparison of the time needed to execute the AI algorithm with different technological platforms and hardware configurations.

Tech. Platform	HW configuration	Patch image (s)	Whole Image (s)
Telemedicine platform	Low-moderate performance GPU (NVIDIA K80)	0.04	1.97
Telemedicine platform	Low-moderate performance CPU (Intel Xeon E5-2630 v3)	0.20	9.60
Mobile-phone	Low-moderate CPU (Snapdragon 820)	0.25	12.00

## 4. Discussion and conclusions

In this work, we present an end-to-end pipeline for microscopy image digitization and remote analysis together with novel deep learning algorithms for automatic assessment and quantification of parasitological infections by STH, mainly focused on the identification of *Trichuris* spp. eggs.

Microscopy remains the most widely used technique to complete a diagnosis of any of the NTDs, including STH which is one among the most prevalent diseases among this group. However, visual diagnosis based on conventional microscopy is a subjective procedure and requires specialised experts in the work field. Unfortunately, the density of health workers in STH-endemic areas is very low [24]. Taking into account these limited resources together with the highly elevated number of patients in these areas, it is clear that the use of digital microscopes together with AI algorithms for remote and automatic diagnosis of STH could constitute an advantageous tool.

Although some systems have been previously proposed for the digitization of STH images, they require special hardware that has not been specifically designed for acquiring microscopy images [17] or could disrupt the usual laboratory workflow since they do not leverage conventional microscopes, making it more complex to follow standard microscope diagnostic protocols [16]. Our 3D printed digitization and image acquisition device was specially designed not to alter the daily routine in microscopy diagnosis laboratories. Additionally, the access to primary care centers in high endemic areas of STH may be limited, entailing a high need for fieldwork to bring the diagnosis closer to remote areas. In this context, we designed a completely portable device with reduced weight and size that can be attached to any existing conventional microscope, enabling digital diagnosis in those areas.

In this work, we proposed the first complete pipeline from image digitization using smartphone technology to remote analysis assisted by AI methods.

The acquisition and digitization of the images were performed in a standardized manner and using a controlled procedure using a customized app which is directly linked to a telemedicine platform where the images were manually analyzed and tagged enabling the training of AI algorithms.

The quality of the ground truth generated by annotating the digitized samples in a remote manner was evaluated and compared to the analysis based on conventional microscopy. The results showed good agreement and minimal differences between the resulting egg quantification using both methods (R-squared of 0.95) and ensured enough ground truth quality to train AI models.

Particularly, the proposed deep learning-based algorithm enables an automatic and objective identification of STH eggs. The method, which was trained and validated using a cross-validation scheme, achieved a relatively high precision and recall results (98,44% and 80,94% respectively) for the identification and classification of *Trichuris* spp. eggs. The results validated the use of the proposed deep learning algorithms for the automatic identification of STH eggs. We also wanted to compare the results with those obtained with more complex and deeper network architectures, and the results proved that the proposed relatively simple architecture considerably reduces computational cost while maintaining similar results.

Additionally, the results obtained suggest that remote and AI-assisted analysis of digitized images of Kato-Katz samples allows to detect in mean more eggs compared to the count using the conventional procedure, since the analysis can be performed in a more exhaustive manner. Moreover, these tools would potentially be useful for other use cases such as the evaluation of the effectiveness of MDA programmes.

For further validation and to illustrate the generalization capability of the method at identifying other helminth eggs than *Trichuri*s spp., we extended the analysis by training the algorithm including positive samples for both *Trichuris* spp. and *Ascaris* spp. coming from a co-infected subject. The results (mean precision of 94,66% and mean recall of 93,08%) showed that the proposed method can be extended for the detection of different STH eggs, although further validation work should be done in this direction.

It should be noted that the main limitation of this work may be caused by the relatively scarce number of considered subjects. This should be contemplated in future works in order to increase the variability regarding STH morphology and stage, appearance and aspect of the Kato-Katz samples and the inclusion of other species of STH (i.e. hookworm).

Finally, we proposed an operational implementation which allows to integrate the AI algorithm on both the remote analysis platform and the digitization mobile app, opening a simple but potentially revolutionary use of the method on demand by invoking it through the telemedicine platform or in real time during image digitization with the mobile app.

Next steps to scale the proposed system in the field require the undertaking of a large-scale clinical performance evaluation study to validate the entire pipeline and demonstrate its applicability where real-time diagnosis is required.

Although effective and accurate molecular tools for the diagnosis of NTDs such as STH have been proposed, which are already implemented in developed countries [25], they may not be accessible in low- and middle-income countries. These molecular techniques, such as quantitative PCR, require expensive equipment and very well-trained specialists. In order to achieve the goal established in the WHO 2030 roadmap [3], it is essential to have an effective and standardized diagnosis to accelerate the NTDs elimination. In this context, the proposed solution could reduce time, distances, and expertise needed for microscopic analysis of helminth samples and therefore help to make accurate STH diagnosis accessible.
